# 91 Systemic lupus erythematosus and infectious complications: a pediatric case report

**DOI:** 10.1093/rheumatology/keac496.087

**Published:** 2022-10-07

**Authors:** Djohra Hadef, Badereddine Salhi, Meriem Haddadi, Khaoula Benbrahim, Rimah Belaala, Leila Bouderdaben, Norhane Bensacia, Ibtissam Kahlane, Abdarahmane Abdallaoui, Yacine Khenferi, Nacer Khernane

**Affiliations:** 1 Department of Pediatrics, CHU Batna, Algeria; 2 Faculty de Medicine, University of Batna 2, Algeria; 3 Department of immunology, CHU Batna, Algeria; 4 Department of Orthopedics, CHU Batna, Algeria

## Abstract

**Introduction:**

Infection is one of the leading causes of morbidity and mortality in Systemic Lupus Erythematosus (SLE). Bacterial infections are the most frequent, followed by viral and fungal infections. Therapeutic factors as well as disease- and genetic related factors, all contribute to a lupus patient's increased susceptibility to infections.

This report describes a case of a 12-year-old female who presented with features of systemic lupus erythematosus with inaugural infectious joint involvement.

**Methods and results:**

An 11- year- old girl, from Biskra (Algeria), from a non-consanguineous marriage and the 7th of eight children. She had no relevant past medical history. She was referred to our department for the diagnostic and therapeutic management of a suspected systemic lupus erythematosus.

The girl had a fever with a malar erythema and oral ulcerations. Arthralgia of the right hip was reported. Laboratory tests showed a positive CRP (C-reactive protein) and a positive Procalcitonin. The immunological assessment revealed Anti DNA, Anti Sm and Anti RNP positive.

Ultrasound of the hip showed a very large arthro-synovial cyst of the anterior face of the right hip of 07 cm compressing the psoas muscle. The cyst was drained and antibiotic therapy initiated. Hydroxychloroquine was started while waiting for the rest of the exploration.

**Conclusion:**

Joint involvement in systemic lupus erythematosus is frequent but an infectious complication is possible even at the disease onset and should not be missed.

No conflicts of interest to declare.

